# *Pseudomonas aeruginosa *acquisition on an intensive care unit: relationship between antibiotic selective pressure and patients' environment

**DOI:** 10.1186/cc10026

**Published:** 2011-02-09

**Authors:** Alexandre Boyer, Adélaïde Doussau, Rodolphe Thiébault, Anne Gaëlle Venier, Van Tran, Hélène Boulestreau, Cécile Bébéar, Frédéric Vargas, Gilles Hilbert, Didier Gruson, Anne Marie Rogues

**Affiliations:** 1 Service de Réanimation Médicale, Hôpital Pellegrin-Tripode, place Amélie Raba Léon, 33076 Bordeaux Cedex, France; 2 CHU de Bordeaux, Centre d'Investigation Clinique-Epidémiologie Clinique (CIC-EC 7), Université Victor Segalen Bordeaux 2, 146 rue Léo Saignat, 33076 Bordeaux Cedex, France; 3 Université Victor Segalen Bordeaux 2, Institut de Santé Publique d'Epidémiologie et de Développement (ISPED), 146 rue Léo Saignat, 33076 Bordeaux Cedex, France; 4 INSERM, U897 Epidémiologie et Biostatistiques, 146 rue Léo Saignat, 33076 Bordeaux Cedex, France; 5 INSERM, U657 Pharmaco-Epidémiologie et Evaluation de l'Impact des Produits de Santé sur les Populations, 146 rue Léo Saignat, 33076 Bordeaux Cedex, France; 6 Service d'Hygiène Hospitalière Hôpital Pellegrin-Tripode, place Amélie Raba Léon, 33076 Bordeaux Cedex, France; 7 Service de Bactériologie, Hôpital Pellegrin-Tripode, place Amélie Raba Léon, 33076 Bordeaux Cedex, France

## Abstract

**Introduction:**

The purpose of this study was to investigate the relationship among *Pseudomonas aeruginosa *acquisition on the intensive care unit (ICU), environmental contamination and antibiotic selective pressure against *P. aeruginosa.*

**Methods:**

An open, prospective cohort study was carried out in a 16-bed medical ICU where *P. aeruginosa *was endemic. Over a six-month period, all patients without *P. aeruginosa *on admission and with a length of stay >72 h were included. Throat, nasal, rectal, sputum and urine samples were taken on admission and at weekly intervals and screened for *P. aeruginosa*. All antibiotic treatments were recorded daily. Environmental analysis included weekly tap water specimen culture and the presence of other patients colonized with *P. aeruginosa*.

**Results:**

A total of 126 patients were included, comprising 1,345 patient-days. Antibiotics were given to 106 patients (antibiotic selective pressure for *P. aeruginosa *in 39). *P. aeruginosa *was acquired by 20 patients (16%) and was isolated from 164/536 environmental samples (31%). Two conditions were independently associated with *P. aeruginosa *acquisition by multivariate analysis: (i) patients receiving ≥3 days of antibiotic selective pressure together with at least one colonized patient on the same ward on the previous day (odds ratio (OR) = 10.3 ((% confidence interval (CI): 1.8 to 57.4); *P *= 0.01); and (ii) presence of an invasive device (OR = 7.7 (95% CI: 2.3 to 25.7); *P *= 0.001).

**Conclusions:**

Specific interaction between both patient colonization pressure and selective antibiotic pressure is the most relevant factor for *P. aeruginosa *acquisition on an ICU. This suggests that combined efforts are needed against both factors to decrease colonization with *P. aeruginosa*.

## Introduction

*Pseudomonas aeruginosa *infections on the ICU are a constant concern [1]. Colonization with this organism often precedes infection [2] and its prevention is, therefore, extremely important. *P. aeruginosa *colonization has been reported to originate from exogenous sources such as tap water [3], fomites and/or patient-to-patient transmission, or as an endogenous phenomenon related to antibiotic use. Some studies have highlighted the importance of exogenous colonization during hospitalization (50 to 70% of all colonizations) [4-9] whereas others have questioned its relative importance [10-13]. Molecular epidemiology techniques have given an insight into *P. aeruginosa *acquisition by demonstrating that the same pulsotypes may spread from the environment to patients [14,15], sometimes in an epidemic mode. This could explain the discrepancies between studies with different levels of exogenous acquisition [14-16]. Although genotyping methods are useful, they fail in giving a definitive result for the origin of bacteria. First, a strain shared by a patient and his/her environment has not necessarily been transmitted from the environment to the patient. Furthermore, acquisition of a strain not isolated from the environment does not necessarily mean that it is part of the patient's flora (the classical endogenous definition [17,18]). It could also have been acquired through previous healthcare procedures from undiscovered environmental sources (misdiagnosed exogenous acquisition). Whatever the mode of acquisition, the determinants of colonization remain unclear. In particular, the role of antibiotic selective pressure on *P. aeruginosa *colonization is an important issue.

In a previous study [3], we carried out a genotypic analysis on our medical ICU. This analysis eliminated an exogenous epidemic spread but showed that *P. aeruginosa *colonization was associated with tap water contamination over several weeks. It suggested, together with an overall incidence of 11.3 colonized/infected cases per 100 patients, an endemic *P. aeruginosa *context [3]. However, this study had several limitations. Only genotyping from one colony of each culture was performed so that only one-third of the strains were analysed. Thus, it was not possible to ascertain which acquisition mechanism predominated. More importantly, the potential role of antibiotic selective pressure on acquisition was not studied. Based on the same study population, the aim of the current study was to explore the respective roles of environment and antibiotic selective pressure on *P. aeruginosa *colonization during healthcare delivery in these endemic conditions.

## Materials and methods

### Study setting

**Figure 1:**
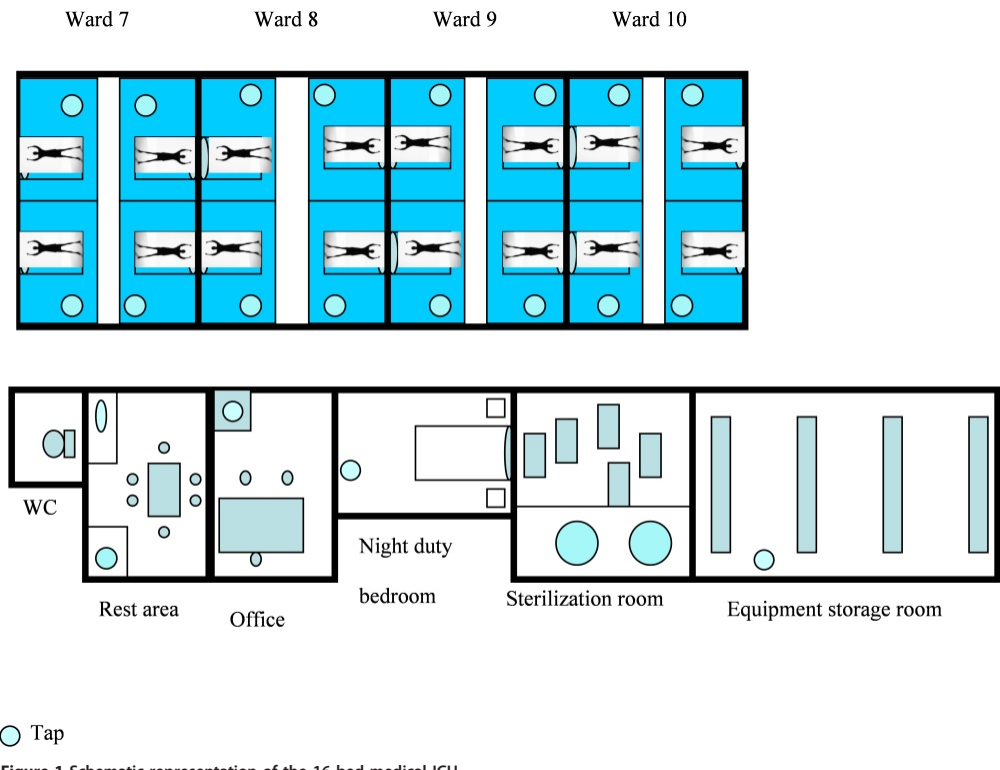
**Schematic representation of the 16-bed medical ICU**.

The study was performed on a 16-bed medical ICU in a 1,624-bed university teaching hospital between April and November 2003 (29 weeks). Patients were treated in single rooms distributed on four wards of four rooms each. Other rooms such as a rest area, sterilization room (a room dedicated to sterilization of medical devices), toilet, equipment storage room, office and night duty bedroom were shared (Figure 1). Each room had its own water tap. The nurse:patient ratio was 1:4. The antibiotic policy and hygiene protocols were not modified during the study period. No digestive decontamination was used on the ICU. Twice monthly chlorine tap water disinfection was started in July (Week 11). Hygiene protocols consisted of contact barrier precautions for medical and nursing staff caring for patients colonized or infected with multi-resistant microorganisms (not including *P. aeruginosa*). These precautions were applied systematically on admission of previously hospitalized patients from other medical or surgical units for more than 48 h and for known carriers. *P. aeruginosa *carriers were identified on admission from rectal and oropharyngeal swabs. No screening was performed at discharge. Hand hygiene procedures were emphasized routinely.

### Patients

All patients admitted during the study period were systematically included in a prospective cohort. Secondary exclusion criteria included: length of ICU stay <72 h and carriage of *P. aeruginosa *on admission. These patients were, however, considered as potential *P. aeruginosa *environmental sources as they were present in the ICU. Data were recorded prospectively each day until *P. aeruginosa *colonization/infection, death, discharge to another unit, or end of the study period. The variables examined for all patients included demographic data (age, gender), underlying conditions (immunosuppression as defined by cancer, AIDS with CD4 T-lymphocytes <100, haemopathy, or corticotherapy >0.5 mg/kg/day, diabetes mellitus, end-stage renal disease, chronic liver disease, chronic heart or respiratory failure) and severity evaluated by the Simplified Acute Physiology Score (SAPS II) [19]. Data regarding the use of intravascular catheters, nasogastric or endotracheal tubes were also collected daily.

This study was approved by our local ethics committee (Comité de Protection des Personnes Sud-Ouest et Outre Mer III, reference number: DC2010/38). The need to obtain informed consent was waived because no change was done to our ICU's usual practices (the endemic context of the ICU justified an intense surveillance procedure), but patients and/or their proxies were informed of the study's purpose.

### Microbiological screening

As a routine surveillance procedure, throat, nasal and rectal swabs as well as sputum and urine samples were collected on admission and weekly thereafter on predefined days. Other specimens were taken when clinically indicated. Environmental screening included weekly tap water samples from the patients' rooms and tap water samples from shared rooms every three weeks. The methods of specimen collection and culture have been described previously [3].

### Definition of acquired *P. aeruginosa *colonization/infection

Acquired colonization/infection was defined as the isolation of *P. aeruginosa *from at least one surveillance or clinical culture from patients not colonized or infected at ICU admission. *P. aeruginosa *infection was defined as a positive culture with clinical and biological manifestations of infection. In cases of lower respiratory tract infection, quantitative cultures were positive if a threshold of ≥10^7 ^colony-forming units (CFU)/ml for tracheal aspirates or ≥10^4 ^CFU/ml for bronchoalveolar lavage were obtained.

### Risk factors for *P. aeruginosa *colonization/infection

#### Antibiotics

Antibiotic treatment was recorded daily and classified according to *P. aeruginosa *susceptibility (no antibiotic treatment, inactive or active against *P. aeruginosa *including ureido and carboxypenicillins, antipseudomonal cephalosporins, carbapenems, fluoroquinolones, aminoglycosides, colimycin, fosfomycin). If a patient was treated simultaneously with both active and non-active antibiotics, the patient was considered to have been treated with active antibiotics.

#### Environmental factors

Systematic environmental screening included other patients from the ward on which the patient was hospitalized, other patients on the ICU, tap water from the same ward, tap water from the ICU and tap water from shared rooms. Daily indices of environmental pressure were calculated as assessed in other studies of patient-induced colonization pressure [11]. Briefly, for each study day, the number of patients and tap water samples colonized with *P. aeruginosa *on the ward/ICU where the patient was hospitalized was estimated. Two variables were then described: (i) the colonization of patients or tap water samples on the previous day (called previous patient/tap water colonization pressure); and (ii) the number of patients or tap water samples colonized since the patient's admission (called cumulative patient/tap water colonization pressure). Environmental exposure was assumed to be constant between two screenings. Hence, patients who acquired *P. aeruginosa *had several environmental pressure profiles (including patient colonization pressure and tap water colonization pressure) allowing a comparison with patients who did not acquire *P. aeruginosa*.

### Statistical analysis

Quantitative variables were compared using the Student's *t*-test or Wilcoxon test according to the distribution of data. Qualitative variables were compared using the Chi^2 ^or Fisher's exact test. A marginal logistic regression model accounting for repeated measurements [20] was used to assess the relationship between environment, antibiotic pressure and *P. aeruginosa *acquisition each day, and the results were expressed as odds ratios (OR) and 95% confidence intervals (CI). Univariate analysis of *P. aeruginosa *acquisition included: (i) fixed variables for patient characteristics at admission; (ii) longitudinal data on patient/tap water colonization pressures, as described above, on the cumulative number of days since admission with a nasogastric tube (which was selected to represent invasive devices as it is strongly associated with the use of other invasive devices in our clinical practice) or with antibiotics classified as active or inactive against *P. aeruginosa. *Selection of the environmental exposure index (previous or cumulated colonization pressure) was based on Akaike criteria [21]: patient/tap water colonization pressure on the previous day was finally introduced in the multivariate analysis. Quantitative data were analyzed as categorical variables when the log-linearity assumption was not followed. All factors with a *P-*value < 0.20 in univariate analysis were selected for multivariate analysis. In multivariate analysis, the factors related to patient/tap water colonization pressures, that is, "patients on the same ward", "tap water from the ICU", "tap water from the shared rooms" or antibiotics were first introduced together and forced in the model. Because wards are included in the ICU, only the most significant index among colonization pressure onto the ward or the ICU was selected for analysis purpose. Other factors were then introduced in a stepwise manner to control for confounding. According to our main objective, the final model looked for interactions between each of the three patient/tap water colonization pressures and antibiotic variables. A *P-v*alue of <0.05 was considered significant. Data were recorded prospectively with Epidata (3.1; Odense, Denmark). The model was fitted using the GENMOD procedure on SAS software (SAS Institute, Inc., Cary, NC, USA).

## Results

### Study population

Of the 415 patients admitted to the ICU during the 29-week study period, 262 were excluded because their length of stay was <72 h and 27 were excluded because screening at admission revealed *P. aeruginosa*. Finally, 126 patients were included, comprising 1,345 patient-days. The demographic and clinical characteristics of these patients are shown in Table 1.

**Table 1:** Demographic and clinical characteristics of the study population (*n *= 126)

Characteristic	
Age (years)	57 ± 17
Male/female	72/54
SAPS II	45 ± 18
Hospitalization before admission	88 (70.0%)
Underlying conditions	0.7 ± 0.7
immunosuppression	29 (23.0%)
chronic respiratory failure	24 (19.0%)
diabetes	22 (17.5%)
heart disease	4 (3.2%)
renal disease	4 (3.2%)
cirrhosis	2 (1.6%)
Invasive device	
Mechanical ventilation (%)	78
Duration (days)	6 (2 to 10)
Central venous catheter (%)	65
Duration (days)	5 (0 to 10)
Nasogastric tube (%)	72
Duration (days)	6 (0 to 10)
Enteral nutrition (%)	93
Duration (days)	6 (4 to 9)
Foley catheter (%)	79
Duration (days)	6 (2 to 11)
Length of stay (days) median	8 (6 to 12)
ICU mortality	29 (23%)

Values are shown as mean ± SD, n (%), or median (1^st ^to 3^rd ^quartile).

SAPS II: Simplified Acute Physiology Score; ICU: intensive care unit.

### Microbiological screening

During the study, microbiological screening yielded 807 samples: 166 sputum or bronchoalveolar cultures, 144 blood cultures, 114 nasal, 111 rectal, 109 throat, 108 urine and 55 miscellaneous cultures. Cultures were not available for 15 patients, accounting for 94 patient-days. Each patient had a median of five cultures (range: two to nine) during their ICU stay. Acquired *P. aeruginosa *was present in 27 cultures (3.4%): 11 respiratory, 7 rectal, 4 throat and 3 nasal cultures, 1 stool and 1 peritoneal sample.

### Acquired colonization/infection

Twenty patients (16%) acquired *P. aeruginosa *during their ICU stay. *P. aeruginosa *colonization was present in 11 patients: rectal culture (*n *= 5), sputum culture (*n *= 2), rectal and throat or nasal culture (*n *= 2), sputum culture associated with rectal, nasal and throat colonization (*n *= 1) and stool culture (*n *= 1). *P. aeruginosa *infection was observed in nine other patients (nosocomial pneumonia (*n *= 8) and nosocomial peritonitis (*n *= 1)). *P. aeruginosa *isolation occurred a median of 11 days (range: 8 to 16) after admission.

### Antibiotic treatment

During their ICU stay, 106 patients (84%) received a total of 970 antibiotic days with a median of two antibiotics (range: one to three) for a median duration of seven days (range: 3 to 11) per patient. The antibiotics used are described in Table 2. All patients who acquired *P. aeruginosa *(except one) had received antibiotics before acquisition (median of two antibiotics (two to four) vs. median of two antibiotics (two to three) in the other group; *P *= 0.09). Among the 106 patients treated with antibiotics, two-thirds (*n *= 67) received at least one day of antibiotics active against *P. aeruginosa *whereas one-third (*n *= 39) did not.

**Table 2:** Distribution of antibiotic treatment according to acquisition group*

	*P. aeruginosa *acquisition *n *= 20 (%)	No *P. aeruginosa *acquisition *n *= 106 (%)	Total *n *= 126 (%)
**Antibiotics active against *P. aeruginosa***	**10 (50)**	**57 (54)**	**67 (53)**
Aminosides	6 (30)	17 (16)	23 (18)
Ureido/carboxypenicillins	5 (25)	19 (18)	24 (19)
Piperacillin-tazobactam	5 (25)	12 (11)	17 (13)
Ticarcillin-clavulanic acid	0 (0)	7 (7)	7 (6)
Antipseudomonal cephalosporins	3 (15)	13 (12)	16 (13)
Ceftazidime	3 (15)	6 (6)	9 (7)
Cefepime	0 (0)	7 (7)	7 (6)
Carbapenems	4 (20)	12 (11)	16 (13)
Fluoroquinolones	7 (35)	33 (31)	40 (32)
Others	1 (5)	3 (3)	4 (3)
Fosfomycin	0 (0)	2 (2)	2 (2)
Colomycin	1 (5)	1 (1)	2 (2)
**Antibiotics not active against *P. aeruginosa***	**14 (70)**	**85 (80)**	**99 (79)**
Glycopeptides	5 (25)	30 (28)	35 (28)
Non-antipseudomonal penicillins	4 (20)	43 (41)	47 (37)
Penicillin G	0 (0)	1 (1)	1 (1)
Penicillin M	0 (0)	2 (2)	2 (2)
Amoxicillin	1 (5)	3 (3)	4 (3)
Amoxicillin-clavulanic acid	3 (15)	37 (35)	40 (32)
Non-antipseudomonal cephalosporins (cefotaxim; cefuroxim; ceftriaxon)	10 (50)	23 (22)	33 (26)
Macrolides	5 (25)	12 (11)	17 (13)
Other	2 (10)	18 (17)	20 (16)
Pristinamycin	0 (0)	3 (3)	3 (2)
Metronidazole	0 (0)	10 (9)	10 (8)
Cotrimoxazole	1 (5)	1 (1)	2 (2)
Rifampicin	1 (5)	4 (4)	5 (4)

* The data represent the number of patients who received at least one day of antibiotic of each class (percentage of patients in each group).

### Environmental screening results

The results of environmental screening are shown in Table 3. In addition to the 20 patients who acquired *P. aeruginosa *during the study, 27 patients were colonized and/or infected with *P. aeruginosa *at ICU admission. Thus, 47 patients potentially contributed to the patient colonization pressure. Tap water screening from the patient's rooms yielded 152/464 positive samples (33%). Surveillance of tap water from shared rooms yielded 72 samples, of which 12 were positive for *P. aeruginosa *(17%). Contaminated tap water was observed four times in the shared toilet, three times in the sterilization room, twice in the night duty bedroom and once in the rest area, office or equipment storage room. The implementation of tap water disinfection at Week 11 of the study should have decreased the patients' environmental pressure. However, no significant interaction was found between tap water colonization and time period (before or after Week 11) (*P *= 0.69).

**Table 3:** Summarization of environmental screening data according to acquisition group

	*P. aeruginosa *acquisition (*n *= 20)	No *P. aeruginosa *acquisition (*n *= 106)	Total (*n *= 126)
Cumulative patient-induced environmental pressure*			
From the same ward	1.2 (0.6 to 1.8)	0.8 (0 to 1.7)	1 (0.1 to 1.8)
From the ICU	4.8 (3.6 to 5.6)	4.7 (3.3 to 5.6)	4.7 (3.3 to 5.6)
Cumulative tap water-induced environmental pressure*			
From the patients' wards	0.1 (0 to 0.7)	0 (0 to 0.6)	0 (0 to 0.6)
From the ICU	1.9 (1.1 to 2.3)	1.6 (0 to 3)	1.8 (0 to 2.9)
From shared rooms	1 (0.7 to 2.3)	0.8 (0 to 1)	1 (0 to 1)
Patient-induced environmental pressure**			
≥1 colonized patient on the same ward			
yes	20	79	99
no	0	27	27
≥1 colonized patient on the ICU			
yes	20	106	126
no	0	0	0
Tap water-induced environmental pressure**			
≥1 colonized tap water on the same ward			
yes	10	51	61
no	10	55	65
≥1 colonized tap water on the ICU ^¤^			
yes	18	68	86
no	2	38	40
≥1 colonized tap water in shared rooms			
yes	17	70	87
no	3	36	39

Values shown are: median (1^st ^to 3^rd ^quartile), or n.

*Cumulative patient/tap water-induced environmental pressure represents the number of contaminated patients/tap water samples since admission.

**Patient/tap water-induced environmental pressure represents the number of patient that were exposed to a contaminated patient/tap water at least one time during their ICU stay.

^¤ ^Excluding tap water in shared rooms.

### Risk factors for *P. aeruginosa *acquisition

By univariate analysis, the presence of an invasive device (nasogastric tube), previous patient colonization pressure on the same ward and previous tap water colonization pressure from the ICU and shared rooms were significantly associated with *P. aeruginosa *acquisition (Table 4). Multivariate analysis revealed that the presence of a nasogastric device was independently associated with *P. aeruginosa *acquisition (OR = 7.72 (95% CI: 2.32 to 25.70); *P *= 0.001). In addition, the interaction between antibiotics inactive against *P. aeruginosa *and the patient colonization pressure was also significant (*P *< 0.03). It means that, in patients receiving equal to or more than three days of antibiotics inactive against *P. aeruginosa*, the presence of at least one colonized patient on the same ward on the previous day increased the risk of *P. aeruginosa *acquisition on a given day (OR = 10.26 (95% CI: 1.83 to 57.43); *P *= 0.01) compared to patients without colonized patient in the same ward. This association was not observed in patients with less than three days of antibiotics inactive against *P. aeruginosa.*

**Table 4:** Risk factors for *P. aeruginosa *acquisition in the ICU (*n *= 126)

	Univariate analysis		Multivariate analysis	
		
Risk factor	OR (95% CI)	*P*	OR (95% CI)	*P*
SAPS II				
≥43 (vs. <43)	2.54 (0.89 to 7.24)	0.08	*	
Age				
≥70 years (vs. <70)	4.61 (1.67 to 12.72)	0.14	*	
Nasogastric tube				
Equal to or more than nine cumulated days since admission (vs. less than nine days)	7.66 (2.88 to 20.36)	<0.0001	7.72 (2.32 to 25.70)	0.001
Antibiotic treatment not active against *P. aeruginosa*				
More than three days (vs. zero to two days)	2 (0.76 to 5.27)	0.16	***	
Antibiotic treatment active against P. aeruginosa**				
per cumulated day since admission	1.02 (0.95 to 1.10)	0.54	****	
Previous patient-induced environmental pressure				
Equal to or more than one colonized patient on the same ward on the previous day (vs. zero)	4.91 (1.47 to 16.39)	0.01	***	
Equal to or more than one colonized patient on the ICU on the previous day (vs. zero)	1.14 (0.27 to 4.90)	0.86	****	
Previous tap water-induced environmental pressure				
Equal to or more than one colonized tap water on the same ward on the previous day (vs. zero)	2.37 (0.96 to 5.89)	0.06	^$^	
Equal to or more than one colonized tap water on the ICU on the previous day (vs. zero)	3.79 (1.26 to 11.44)	0.02	1.99 (0.67 to 5.88)	0.21
Equal to or more than one colonized tap water in shared rooms on the previous day (vs. zero)	4.63 (1.37 to 15.65)	0.01	3.07 (0.93 to 10.16)	0.07
Interaction between previous patient-induced environmental pressure and inactive antibiotics:				0.03^$$^
If equal to or more than three days of inactive antibiotics			1	
- no colonized patient on the same ward on the previous day			10.26 (1.83 to 57.43)	0.01
- equal to or more than one colonized patient on the same ward on the previous day				
If zero to two days of inactive antibiotics				
- no colonized patient on the same ward on the previous day			1	
- equal to or more than one colonized patient on the same ward on the previous day			1.00 (0.26 to 3.87)	0.99

*Factors removed by stepwise forward procedure.

**Log-linearity was assumed for this factor.

***Factors included in the interaction.

****Not statistically eligible by univariate analysis (*P *> 0.20).

^$^Not included in multivariate analysis for colinearity with tap water in the ICU.

^$$^*P *for overall interaction.

## Discussion

This study suggests two main conclusions. First, *P. aeruginosa *acquisition should be related to the proximity of a patient colonized with *P. aeruginosa *in the area (same room) with a chronological component (the previous day) along with selective antibiotic pressure. Antibiotic selective pressure alone did not influence *P. aeruginosa *acquisition. The hypothesis of a complex mechanism involving antibiotic selective pressure and patient colonization pressure should be relevant for *P. aeruginosa *acquisition in an ICU with endemic context. If the interaction of both pressures overriding each pressure taken separately is reviewed, there could be some practical implications. Developing strategies for either decreased antibiotic use for "endogenous-like" acquisition or hygiene improvement in response to environmental contamination in "exogenous-like" acquisition could be insufficient. In an endemic ICU without obvious epidemic acquisition, it is arguable that a reduction in antibiotic selective pressure and improvement in hygiene standards should be combined. The second conclusion is that invasive devices remain an important determinant in *P. aeruginosa *acquisition. Whether invasive devices are a surrogate of patient's severity (an already known acquisition risk factor) or a step for bacteria in the chain linking the environment to the patients cannot be inferred from the results of this study.

In our study, the classical binary endogenous/exogenous scheme [12,22] is transcended by the interaction of both factors, which confirms that *P. aeruginosa *acquisition is complex. In the past, some molecular epidemiology studies have reported a significant role of exogenous colonization [4-7,18], whereas others have predominantly identified the role of endogenous colonization [11,13]. Genotypic methods may detect an epidemic context where exogenous sources are the most important [23] and potentially overestimate its role. Hence, the same group has described two different levels of exogenous *P. aeruginosa *cross-transmission [9,11]. It is also likely that strains spread rapidly from patients to the environment and vice-versa, complicating environmental and patient screening because screening at distinct time intervals could misclassify some cases of exogenous acquisition [16]. Special attention should also be paid to so-called "endogenous" *P. aeruginosa *acquisition. *P. aeruginosa *is not generally considered to be part of the normal human flora [16], and in most patients admitted to hospital for the first time, *P. aeruginosa *is not usually isolated from bacteriological specimens until the patient has been in the hospital for several days [22,24,25]. In these cases it is unclear if *P. aeruginosa *is really endogenous (that is, present on admission but undetected by screening and only revealed by antibiotic selective pressure) [17,18]. On the other hand, despite being absent from the flora on admission, *P. aeruginosa *could be acquired from the environment through repetitive daily healthcare procedures. Sequential cultures with *P. aeruginosa *isolation from oropharyngeal samples before the gastrointestinal tract support this hypothesis [26]. Moreover, Johnson *et al. *[22] recently observed that 50% of imipenem-resistant *P. aeruginosa *acquisition corresponded to neither the classical endogenous nor exogenous route. The question of an undiscovered environmental source was raised. This is the case in some endemic ICU contexts [27]. In our ICU the endemic context was suggested by the fact that one-third of the strains shared the same genotypic profile without an obvious exogenous source of acquisition or epidemic profile [3].

Irrespective of the obvious, undiscovered exogenous or true endogenous source of *P. aeruginosa *[28], it is likely that acquisition of this microorganism by patients is related to a third factor, namely antibiotic treatment which could interact with the environment to facilitate *P. aeruginosa *acquisition. Our study confirms this hypothesis. It focused on individual patients with daily recorded antibiotic treatment rather than on a population with collective consumption data [29]. Daily antibiotic recording does not prevent misclassification of antibiotic treatment as active, whereas it was eventually inactive due to poor PK/PD optimization. Even if there is still poor knowledge of the optimal antibiotic dosing strategies to prevent the selection of resistance, an antibiotic stewardship designed to limit insufficient antibiotic doses was set up at the study period, potentially limiting this bias. Besides, all previously known risk factors were adjusted for, as well as widespread and repeated patient and tap water screening (including samples from shared rooms), which have not always been completely (only patient-to-patient transmission) [11,18] or properly (type and frequency of environmental screening) [10,13] assessed. Moreover, active antibiotics were distinguished from inactive antibiotics (selective antibiotic pressure), which could help *P. aeruginosa *become dominant in the patients' flora.

In our ICU, as potentially in others with the same endemic and antibiotic consumption profiles, the results of this study will lead to the development of coordinated strategies against the use of antibiotics that are inactive against *P. aeruginosa *(such as a decrease in systematic penicillin or cephalosporin treatment for aspiration pneumonia) and against the environmental spread of bacteria. The latter should include alcohol-based hand-cleaning programmes since cross-contamination between patients and contaminated tap water was suspected in our study. Contaminated tap water and patients' samples were associated with *P. aeruginosa *acquisition in univariate analysis but only patients' samples were significant in multivariate analysis. Positive cultures from shared rooms were associated with *P. aeruginosa *acquisition in univariate analysis and should be interpreted as additional to ICU *P. aeruginosa *colonization pressure.

There are several limitations to our study. It was a single-centre study and the limited observations may give reduced power to detect other contributing risk factors. These limitations prevent its application to other ICUs where the patient case mix, prevalence of *P. aeruginosa *colonization at admission and antibiotic consumption are different. Antibiotic selective pressure could have played a role in revealing a pre-existing *P. aeruginosa *flora shared with the patient's environment without a cause-and-effect relationship (which would only have been demonstrated by chronological acquisition of the same genotypic strain) or in rendering the patient susceptible to *P. aeruginosa *acquisition from the environment. Other limitations include the fact that adherence to hygiene rules was not assessed, antibiotic consumption before admission was not recorded and *P. aeruginosa *screening was not performed at the end of the ICU stay. Moreover, the environment (patients and tap water) was screened by intermittent samples. However, the inclusion in the model of the most recent sample provided a closer analysis of the time-dependent process of acquisition. Finally, routine surveillance cultures were not obtained from 15 patients with a short stay, although this probably did not significantly influence our findings as they accounted for only 7% of total patient-days.

## Conclusions

In conclusion, this study adds further support for an interaction between the patient colonization pressure and antibiotic selective pressure in the process of *P. aeruginosa *acquisition in the ICU. These results should be confirmed in a larger study in order to generalize their potential implications (that is, target strategies aimed at decreasing antibiotic treatment, where possible, and improving hygiene protocols).

## Key messages

• *Pseudomonas aeruginosa *is still a leading cause of nosocomial infections, yet its mode of acquisition remains the subject of debate.

• In a given patient, the interaction between the environment and the selective antibiotic treatment he (she) just received deserves more study.

• This single-centre ICU-based study shows that a specific interaction between both patient colonization pressure and selective antibiotic pressure is the most relevant factor for *P. aeruginosa *acquisition.

• Prevention of acquisition in a given patient should include both antibiotic stewardship and cross-transmission prevention.

## Abbreviations

AIDS: Acquired Immunodeficiency Syndrome; CFU: colony-forming units; CI: confidence interval; ICU: intensive care unit; OR: odds ratio; *P. aeruginosa*: *Pseudomonas aeruginosa*; PK/PD: pharmacokinetic/pharmacodynamic; SAPS II: Simplified Acute Physiology Score.

## Competing interests

The authors declare that they have no competing interests.

## Authors' contributions

AB conceived the study, participated in its design and in acquisition of data, coordinated the study and wrote the article. AD participated in the design of the study, performed the statistical analysis, participated in the article redaction, and contributed to this study equally with AB. RT participated in the design of the study and coordinated the statistical analysis. AGV participated in the design of the study. VT carried out the acquisition of data. HB participated in the environmental acquisition of data. CB coordinated the bacteriological study. FV participated in the acquisition of patients' data and in the conception of the study. GH participated in the conception of the study. DG conceived the study, participated in its design and in the article redaction. AMR conceived the study, participated in the environmental acquisition of data, in its design and in the article redaction.
